# Minimum standards for evaluating machine-learned models of high-dimensional data

**DOI:** 10.3389/fragi.2022.901841

**Published:** 2022-09-13

**Authors:** Brian H. Chen

**Affiliations:** ^1^ FOXO Technologies Inc, Minneapolis, MN, United States; ^2^ The Herbert Wertheim School of Public Health and Human Longevity Science, University of California San Diego, La Jolla, CA, United States

**Keywords:** aging, epigenetics, epigenetic clock, DNA methylation, machine learning, reproducibility, validation, omics

## Abstract

The maturation of machine learning and technologies that generate high dimensional data have led to the growth in the number of predictive models, such as the “epigenetic clock”. While powerful, machine learning algorithms run a high risk of overfitting, particularly when training data is limited, as is often the case with high-dimensional data (“large *p*, small *n*”). Making independent validation a requirement of “algorithmic biomarker” development would bring greater clarity to the field by more efficiently identifying prediction or classification models to prioritize for further validation and characterization. Reproducibility has been a mainstay in science, but only recently received attention in defining its various aspects and how to apply these principles to machine learning models. The goal of this paper is merely to serve as a call-to-arms for greater rigor and attention paid to newly developed models for prediction or classification.

## Introduction

In recent years, the number of publications describing machine learning models for the estimation of chronological and biological age have risen dramatically. The most well-known example is that of the “epigenetic clock,” although models have also been developed using transcriptomics, miRNA, proteomics, and clinical phenotypes (Peters et al., 2015; Horvath and Raj 2018; Huan et al., 2018; Tanaka et al., 2018; Sun et al., 2021). Here, we define a “model” as a specific algorithm that uses a specific set of input variables (*e.g.*, DNA methylation markers) to estimate a specific output (*e.g.*, chronological age). The current trend in constructing these models or “algorithmic biomarkers” utilizes machine learning methods, but more primitive algorithmic “scores” have existed for decades. (Matthews et al., 1985; Wilson et al., 1998).

Algorithmic biomarkers differ from conventional biomarkers in that they consist of mathematical calculations often from multiple markers rather than a single physical marker that can be observed directly. Determining the parameters of each model requires a “training” dataset in which the model parameters are optimized to fit the data (*e.g.,* minimizing the squared error in a regression). The field of machine learning involves a diverse set of approaches that seek to identify patterns and develop models that fit a set of data. However, advances in computing power, the ability to generate large amounts of data, and the efficiency of machine learning algorithms can lead to highly complex models that fit the training data too well, such that the model does that generalize to independent samples (*i.e.*, poor out-of-sample performance). Thus, the need for a machine-learned model’s performance to be reproduced in multiple studies is of even greater importance than other realms of science.

The growing ease of computing, generating high dimensional data, and data sharing, in combination with current trends in the development of “second generation epigenetic clocks” and clocks in non-human species, lead one to surmise that the number of machine learning models will continue to increase (Levine et al., 2018; Lu et al., 2019; Belsky et al., 2020; Arneson et al., 2022). Furthermore, the number of potential models that could be created are immense. Entirely different models can be developed with even the most subtle of changes that vary combinations of inputs, tuning parameters, study populations, and multiple other factors in the training data. The resulting number of possible models for a single outcome, such as chronological age, may eventually eclipse the number of original inputs used to build the models. This can lead to confusion in the field as to where to focus research efforts.

**FIGURE 1:**
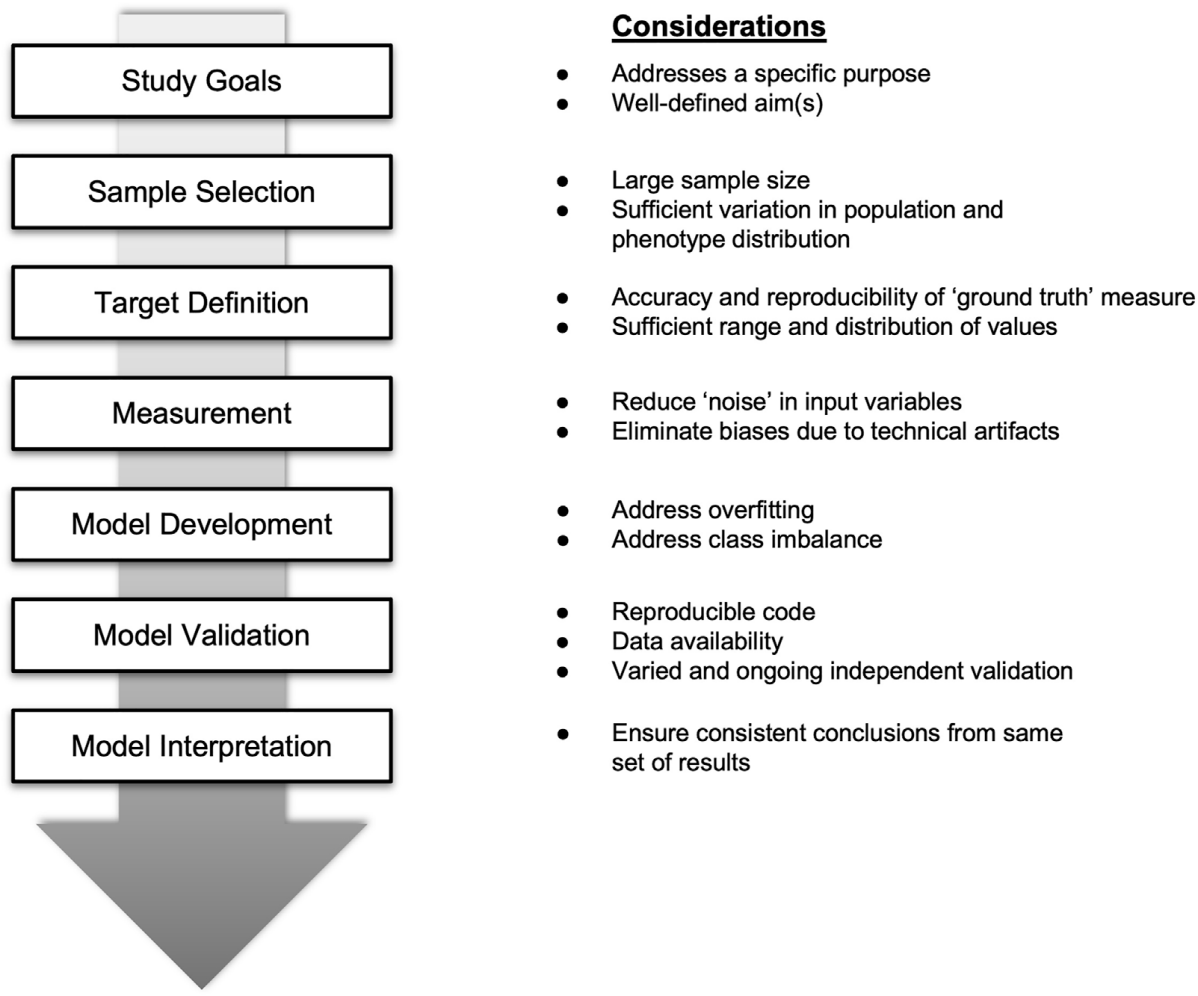
Key considerations for evaluating and enhancing the performance of prediction or classification models using high dimensional data.

Thus, there is a need to raise the bar for reproducible models that have been replicated in independent datasets at the outset, as is common in fields such as genomics (Kraft, Zeggini, and Ioannidis 2009). It should be noted that reproducibility occurs at multiple levels—from development to validation of models (Figure 1). Moreover, greater efforts are also needed to better characterize available models to understand their robustness across different contexts, such as in different study populations.

### Purpose-driven creation of machine learning models

Scientific innovation has always been driven by identifying gaps in knowledge and designing studies to fill those gaps. However, one unintended consequence of advances in generating large amounts of data and increased computing efficiency has been the relative ease and appeal of developing machine learning models using any available data. In some cases, available data included measures that were unique to the original dataset, thereby making independent validation of the model inherently challenging (Sun et al., 2021).

Scientific advances can occur at multiple levels from gaps in knowledge to performance improvements. Gaps in knowledge can include whether a specific set of plasma proteins can estimate an individual’s bone mineral density or risk of osteoporosis. Including this set of proteins in one’s model may boost performance. But gaps in performance of existing models may also serve practical purposes and provide reason to develop new models with specific characteristics, even if the input variables remain the same. Factors such as model accuracy, technical reproducibility, cost, and existing patents and licenses may dictate certain criteria that a specific model must meet. For example, in cases where a diagnostic test is burdensome and/or expensive (e.g., tumor biopsy), developing an inexpensive but highly sensitive screening test may be desirable (e.g., circulating tumor DNA). Models designed to fit a practical need often helps the researcher to articulate the specific parameters and problem(s) that the model will attempt to address.

Notable examples of improvements in existing epigenetic clocks included second-generation epigenetic clocks that were designed to capture physiological aging (rather than chronological age). Other clocks addressed a clear gap in the field by using methodological approaches that employed principal components analysis and restricting inputs to technically reproducible probes (Higgins-Chen et al., 2021; Sugden et al., 2020). Model descriptions that clearly communicate their distinct contribution to the field can help identify the specific use-case for each new model.

### Multi-layered reproducibility

Goodman et al. made the distinction between “truth” and “reproducibility,” where the latter is foundational to the former, but both are distinct notions (Goodman, Fanelli, and Ioannidis 2016). Without reproducible science, it would be difficult to draw any conclusions that move us closer to any truths. Highly reproducible findings are more likely to be true. Less reproducible findings call into question—but do not necessarily rule out—whether a finding is true or not.

In the grand view of reproducibility described by Goodman et al., there exist three distinct layers for reproducibility to occur for a given study—(1) methods reproducibility, (2) results reproducibility, and (3) inferential reproducibility (Goodman, Fanelli, and Ioannidis 2016). Methods reproducibility is the ability to reproduce the results given the same conditions, methods, analytical datasets, and codes used by the authors. In the field of machine learning this is sometimes referred to as technical reproducibility or computational reproducibility (Heil et al., 2021; McDermott et al., 2021).

Methods reproducibility can be enhanced by practices such as providing detailed descriptions of one’s methods, making analytical datasets available for independent verification, making available software codes and detailed instructions, including descriptions of the computing environment, and to make available entire workflows that make verification simple (Heil et al., 2021). Other ideas that have been proposed to enhance methods reproducibility include taking advantage of novel data licenses, privacy-preserving analytic frameworks, and co-authorship to investigators tasked strictly for validation (Peng, Dominici, and Zeger 2006). Moreover, leveraging a differential privacy framework can preserve anonymity of individual data by introducing a predetermined amount of “fake data,” which can then be accounted for when training models (Heil et al., 2021).

Results reproducibility involves reproducing the same findings after following the same experimental methods but using independently generated data. The notion of results reproducibility is often used interchangeably with replicability, external validation, or independent validation. Oftentimes, results reproducibility is driven by the availability of certain datasets, which often differ in a multitude of ways. In an ideal world, validation should be evaluated in many large datasets, each differing from the other in only one distinct way whether it be in a certain measure, methodology, population, or ground truth measure. Such a setup allows a model to be evaluated across a range of contexts to identify characteristics and limitations of a model that one could not evaluate by looking at any single dataset alone. This would be akin to identifying sources of heterogeneity in a large meta-analysis, except the goal would be to identify factors driving differences in performance, if any. Adopting an ongoing approach to validating models can help identify the performance, robustness, and limits of specific models. Only through understanding the strengths and weaknesses of each model can we begin to understand how, when, where, and for whom each model should be used.

In practice, validation datasets may differ from the training dataset in many ways, so a lack of replication should not lead one to automatically discount the model being evaluated since, oftentimes, not all experimental methods were followed. As an example, a predictive mortality model designed for patients in an assisted living home would not be applicable to a young adult population of military soldiers, as both the population and their immediate risks differ dramatically. That said, a failure to independently validate a model, while not entirely conclusive, does provide some evidence as to the robustness of a model. In other words, the model does not work in at least one specific context. In contrast, successful validation in an independent dataset, however large the sample size, is rarely definitive. Each independent validation study is but a single stone in building a wall of evidence supporting the results reproducibility of a model.

Validation can also be improved at the model-building level by utilizing techniques that may enhance model accuracy or reproducibility. To date, biological age estimators using high dimensional data have included DNA methylation, miRNA, and proteomics (Horvath 2013; Huan et al., 2018; Levine et al., 2018; Tanaka et al., 2018). Detailed descriptions of the unique challenges of the various technological platforms are beyond the scope of this paper, but each are well described elsewhere (Lappalainen and Greally, 2017; Joshi and Mayr, 2018) Sugden et al. demonstrated that improving the technical reproducibility of the input variables may also improve the reproducibility of the model outputs (Sugden et al., 2020). Higgins-Chen et al. recently demonstrated that principal components can be used to improve the reliability of specific epigenetic clocks (Higgins-Chen et al., 2022). Thus, models can differ in performance even with the same training data and initial sets of inputs.

The need for extensive, ongoing independent validation across a multitude of datasets is even more essential with machine learning models. Several notable examples exist where machine learning algorithms captured unintended artifacts of the data. One example of a machine learning model designed to discriminate images of huskies from wolves was found to have relied solely on the background of the images of wolves, which tended to be photographed in snowy environments (Ribeiro, Singh, and Guestrin 2016). Another more medically related example was a study of chest x-rays, which were used to build a machine learning model to detect pneumonia only to find that the models were focused on artifacts in the image that denoted which hospital the images were taken (Zech et al., 2018). An extreme example in the aging field may occur when older individuals are selected differently or ascertained differently from younger individuals in the same study, such as in the case of recruiting “healthy agers” across the age range, but not all healthy 20 year-olds end up as healthy 100 year-olds; thus, the selection criteria are not uniform across the age spectrum. Because unintended features may be common across datasets, evaluating models across a wide range of datasets can also help uncover unintended behaviors of models.

It should be noted that the extent to which a validation study follows the methodology of the original experimental protocol is difficult to assess in reality. While conceptually simple, attempts to replicate the methodology of a study are made difficult by a lack of methodological details in the original publication or inability to reproduce every factor in the experimental setup, as there may be many, some that the researcher may have been unaware of, such as the temperature in the laboratory. Similarly, the definition of a successful replication also lacks consensus (Collaboration and Open Science Collaboration 2015). As an example, focusing exclusively on *p*-value “significance” may be misleading if the *p*-value is 0.051 yet the regression coefficients are of similar magnitude and direction in both the original and validation studies. Whether a consensus definition or sufficiently detailed methodological descriptions are achievable or practical, the fact remains that reproducibility is not as black-and-white as one might think.

Lastly, inferential reproducibility refers to the fact that two researchers may interpret the same set of results differently or choose to reanalyze the data differently, both of which may lead to different conclusions than the original author(s). While achieving this level of reproducibility may not be easy, models that achieve this level should be prioritized as they are more likely to reproduce in future studies than models lacking any attempt at reproducibility.

### Ongoing characterization of machine learning models

In addition to the various layers of reproducibility, models that show promise should be further characterized, as is typically required for Clinical Laboratory Improvement Amendments (CLIA) validation of a standard (plasma) biomarker. Aside from data demonstrating the reproducible accuracy of a model against an established ‘ground truth’ measure, the reproducibility of a measure across technical replicates would be valuable. Further investigation could determine through what range of values the model remains accurate and reliable. For instance, an epigenetic clock that relies on or correlates highly with specific blood cell counts may not be relevant in patients undergoing chemotherapy, where blood cell counts may change rapidly and drastically.

Understanding the major sources of variation becomes vital for determining the utility of machine learning models that utilize biological data. However, variation is introduced at multiple levels and multiple sources. Technical variation across replicate samples can describe the reliability of a biological assay. Technical variation can be further subdivided into within-batch and between-batch variation, and “batches” can be subdivided further across different levels from laboratory, days, or certain steps in a protocol.

Another source of variation unique to machine learning models is model variation. This is often due to noise in the training data or the use of randomness in the algorithms (*e.g.*, splitting/shuffling data or initializing parameters). While both technical and model variation are undesirable, a third source of variation can be informative.

Biological variation includes circadian rhythms and the natural biological response to intrinsic and extrinsic forces leading to variation in a biomarker. Understanding the major sources of biological variation in a biomarker provides insights into its proper use and interpretation. As an example, plasma insulin levels respond to food intake, thus fasting levels are needed for proper interpretation in some contexts or, in other contexts, its response to a standardized amount of glucose may be important. Only by understanding the biological sources of variation in a biomarker, even an algorithmic biomarker, can we learn how to utilize and interpret it properly.

Depending on the use-case, one may want to explicitly evaluate whether or not a model is biased against certain subgroups of a population, particularly disadvantaged, underrepresented, and protected classes. Bias, in this context, refers to differences in performance (*e.g.*, accuracy) and not differences in the distributions of model results. To illustrate this point more clearly, men, on average, weigh more than women, so an algorithmic biomarker that estimates age should recapitulate the sex differences in weight. However, the accuracy or reproducibility of the algorithmic biomarker at estimating weight should perform similarly for men and women, separately—or this bias should be accounted for when interpreting results.

The development of benchmarking datasets, such as ImageNet for labeled images, is common in the machine learning field. Since aging clocks are a particular application of machine learning, the development of one or several benchmarking datasets could be helpful. The challenge will be in the data sharing and harmonization of large amounts of data in addition to the relatively limited sample sizes in existing aging cohorts. Because of the large number of variables generated from high dimensional data, the sample sizes needed and commonly found in benchmarking studies would also need to be large.

Clinical trials or longitudinal studies may offer opportunities to demonstrate that model results change within individuals, particularly in ways that one expects in response to known interventions and changes in conjunctions with expected changes in other health or behavioral measures. Completed clinical trials and other datasets with banked biospecimens could be repurposed in such a way as to serve as a resource for rapid validation.

### Discussion

As a call-to-arms was published encouraging replication as a minimum requirement in all genome-wide association studies, the same is needed for machine learning models that utilize “-omic” data (*e.g.*, genomics, epigenomics, phenomics) (Kraft, Zeggini, and Ioannidis 2009). While the multiple layers of reproducibility described above may prove excessive, the bar must be raised beyond where it lies today lest we find ourselves in a world full of unvalidated and poorly characterized models.

As a growing number of consumer products enter the market touting their ability to estimate biological age, independent validation of these model’s accuracy and reproducibility in multiple populations is essential and often lacking. More important may be the transparency, clarity, and accuracy of what is communicated to the consumer about their biological age estimate. Consumers will need help to properly interpret their results.

Contributing to the challenge in proper interpretation or explainability of machine learning models is the double standard to which they are held. Oftentimes, machine learning models are given a large set of inputs whose biological function is not well understood (*e.g.*, DNA methylation levels at 860,000 loci). Then the machine learning algorithms are tasked with finding the optimal set of variables to best fit the data without any requirements for biological plausibility. However, after the fact, the models are unfairly vilified for failing to elucidate any biological mechanisms. While metrics exist to aid in the explainability of a model (*e.g.*, examining the feature weights or SHAP values), the current state of the technology will ultimately fall short of being able to fully explain the biology, but that may be sufficient for certain purposes as long as the models are reproducible in their particular use-case. Machine learning models are designed to predict or classify, and so biological plausibility should be considered a nice-to-have rather than an essential element to demonstrate a model’s validity.

My recommendations can be summarized at a high level. First, developing models with a clear purpose that address a major gap in knowledge can help one’s model stand out. Second, taking great strides to allow the methods to be reproduced with analytic datasets and codes adds to the veracity of the model and, subsequently, its adoption. Further work is needed by journals and professional groups as to how data can be protected yet still allow full vetting of a model. Third, replication of results using independent datasets must be deemed essential for publication and as an ongoing exercise rather than a binary hit-or-miss. Accomplishing these three recommendations provides a step forward in identifying models that may be more likely to be worth the effort of being thoroughly vetted through additional replication and characterization.

Ultimately, the utility of a model is driven by the model’s characteristics and how it will be used. Not all models need to meet the standards of a clinical diagnostic. Most models may still be useful as long as its performance is well documented to enable proper interpretation of its results. Cost, ease of use, accuracy, technical reproducibility, and acceptability by biospecimen donors are some of many factors that are often weighed to determine a model’s utility. However, reproducible findings in independent datasets should be a minimum requirement.
